# Shape-Memory Assisted Scratch-Healing of Transparent Thiol-Ene Coatings

**DOI:** 10.3390/ma12030482

**Published:** 2019-02-04

**Authors:** Algirdas Lazauskas, Dalius Jucius, Valentinas Baltrušaitis, Rimantas Gudaitis, Igoris Prosyčevas, Brigita Abakevičienė, Asta Guobienė, Mindaugas Andrulevičius, Viktoras Grigaliūnas

**Affiliations:** Institute of Materials Science, Kaunas University of Technology, K. Baršausko 59, LT51423 Kaunas, Lithuania; dalius.jucius@ktu.lt (D.J.); valentinas.baltrusaitis@ktu.lt (V.B.); rimantas.gudaitis@ktu.lt (R.G.); igoris.prosycevas@ktu.lt (I.P.); brigita.abakeviciene@ktu.lt (B.A.); asta.guobiene@ktu.lt (A.G.); mindaugas.andrulevicius@ktu.lt (M.A.); viktoras.grigaliunas@ktu.lt (V.G.)

**Keywords:** photopolymerizable, thiol-ene network, scratch-healing, transparent

## Abstract

A photopolymerizable thiol-ene composition was prepared as a mixture of pentaerythritol tetrakis(3-mercaptopropionate) (PETMP) and 1,3,5-triallyl-1,3,5-triazine-2,4,6(1H,3H,5H)-trione (TTT), with 1 wt. % of 2,2-dimethoxy-2-phenylacetophenone (DMPA) photoinitiator. A systematic analytical analysis that investigated the crosslinked PETMP-TTT polymer coatings employed Fourier transform infrared spectroscopy, ultraviolet–visible spectroscopy, differential scanning calorimetry, thermogravimetric analysis, pencil hardness, thermo-mechanical cyclic tensile, scratch testing, and atomic force microscopy. These coatings exhibited high optical transparency and shape-memory that assisted scratch-healing properties. Scratches produced on the PETMP-TTT polymer coatings with different constant loadings (1.2 N, 1.5 N, and 2.7 N) were completely healed after the external stimulus was applied. The strain recovery ratio and total strain recovery ratio for PETMP-TTT polymer were found to be better than 94 ± 1% and 97 ± 1%, respectively. The crosslinked PETMP-TTT polymer network was also capable of initiating scratch recovery at ambient temperature conditions.

## 1. Introduction

Transparent polymer films are widely used as protective coatings in flat panel displays, touch screens, photovoltaic cells, and other devices. Accidental cuts and scratches tend to accumulate on the surface of transparent films and lead to the worsening of the optical transmission and distortion of displayed images. Thus, desirable but still challenging properties of such films are scratch resistance, with an ability to repair the damaged surface by self-healing. 

Nowadays, there is a large variety of self-healing polymers, which can be classified into two broad categories: extrinsic and intrinsic self-healing materials [1,2]. Extrinsic self-healing polymers require inclusion of the specific healing agents that are loaded into microcapsules or vascular networks within a polymeric matrix. In this case, self-healing is triggered by the rupture of healant loaded vessels [3,4,5]. However, fabrication of the highly transparent extrinsic self-healing coatings are complicated, as various inclusions strongly scatter visible light and decrease optical transparency of the films [6]. On the contrary, intrinsic self-healing polymers are able to recover their properties due to the inherent physical interactions, such as molecular interdiffusion or reversible chemical bonds [4]. Reversible chemical bonding includes covalent bonds (i.e., dynamic bond exchange, Diels–Alder reactions, reversible C-ON bonds, photo-reversible reshuffling, and disulfide interchange), non-covalent interatomic bonds (metallic and ionic) and intermolecular forces (hydrogen and Van der Waal’s bonds) [4,7,8]. Exploration of reversible and adaptive noncovalent interactions has resulted in the development of highly complex chemical systems, e.g. supramolecular polymers, which have frequently been employed as self-healing materials [9,10]. Intrinsic self-healing polymers exhibit a latent self-healing functionality that is triggered by damage or by an outside stimulus (e.g. heat, light, or pressure) [11].

Recently, shape-memory polymers (SMPs) have attracted the attention of researchers, as materials capable of recovering their original shape after a temporary deformation when external stimulus is applied provides a mechanism to facilitate self-healing by bringing fractured surfaces into close proximity [12,13,14]. The main advantage of SMPs is the inherent shape recovery effect that eliminates the need for external force to partially or fully close the cracks, scratches, and other surface defects. However, it should be noted that in order to fully heal deep cuts the shape-memory effect is not sufficient and must be combined with other known intrinsic methods of self-healing [7].

A wide variety of polymers have been found to possess shape-memory properties. Among the them are amorphous covalently cross-linked (meth)acrylate-based shape-memory polymer networks prepared using free-radical polymerization, popular for their transparency and tunable properties [15,16]. Main drawbacks of the (meth)acrylate-based polymers include formation of heterogeneous polymer network and inhibition of the polymerization reaction by oxygen. Compared to commonly used (meth)acrylate-based SMPs, thiol-ene polymer systems present numerous advantages, including the negligible oxygen inhibition, low volume shrinkage, homogeneity of the polymer network, toughness, flexibility, and high optical transparency of the polymerized films [17,18,19].

**Scheme 1:**
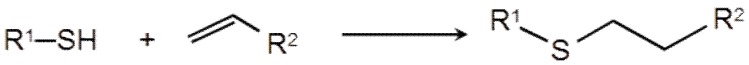
The thiol-ene radical reaction, produced from [20], with permission from © The Royal Society of Chemistry.

**Scheme 2:**
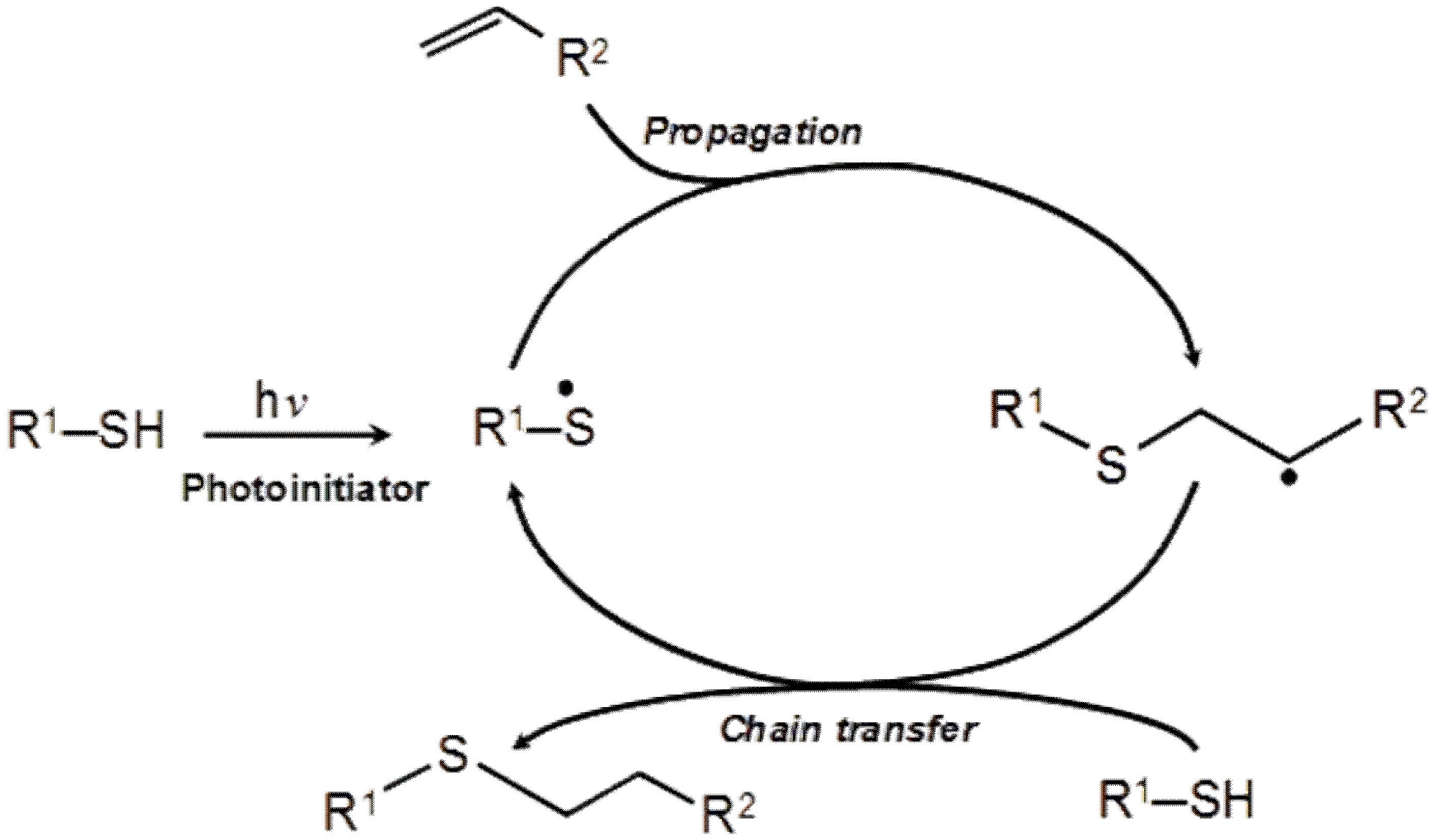
The photochemical radical-initiated step-growth mechanism of the thiol-ene polymerization reaction, produced from [20], with permission from © The Royal Society of Chemistry.

The thiol-ene radical reaction (Scheme 1) is an organic reaction that involves the addition of a thiol to an alkene molecule to form an alkyl sulfide, also referred to as hydrothiolation [20]:

It commonly proceeds through the photochemical radical-initiated step-growth mechanism (Scheme 2), where thiyl radical from thiol component adds across a vinyl functional group of alkene, followed by the hydrogen abstraction from a thiol functional group resulting in the formation of carbon-centered radical, which undergoes chain transfer to a thiol group, regenerating the thiol radical [20,21]. This reaction cycle continues until one component is completely consumed.

Thiol-ene reactions are very fast and can complete in a matter of seconds. Furhter, they can withstand mild reaction conditions, as well as react with a little photoinitiator and can proceed without solvent that can be easily removed. Moreover, they do not require expensive transition-metals as catalysts [22,23,24]. The reaction proceeds more efficiently at pH in the range of 4–7 [25]. Thiol-ene reaction efficiency and kinetics are also highly dependent on the structure of the alkene moiety, where the reactivity is the greatest with strained and electron rich alkenes [26,27,28]. It is one of the most widely used reactions to prepare new crosslinked (e.g. tri- or tetrafunctional thiols and enes) or linear (using dithiols and dienes) branched polymeric structures with relatively narrow glass transition temperature range [29,30,31,32]. A common photoinitiator used in the thiol-ene reactions is 2,2-dimethoxy-2-phenylacetophenone. It gives a benzoyl radical and a tertiary carbon-centered radical which can insert directly into a carbon–carbon alkene bond or abstract a hydrogen atom from a thiol group carbon radical, which starts characteristic thiol-ene free-radical chain reaction [20]. 

Herein, we designed the thiol-ene shape-memory assisted scratch-healing polymer system that responds not only to the temperature changes as the external stimulus but is also capable of initiating a scratch recovery at ambient temperature conditions. Importantly, the developed thiol-ene polymer network exhibits high optical transmittance and holds a great potential as a high-performance flexible transparent material for optoelectronic applications.

## 2. Materials and Methods 

### 2.1. Materials

**Scheme 3:**
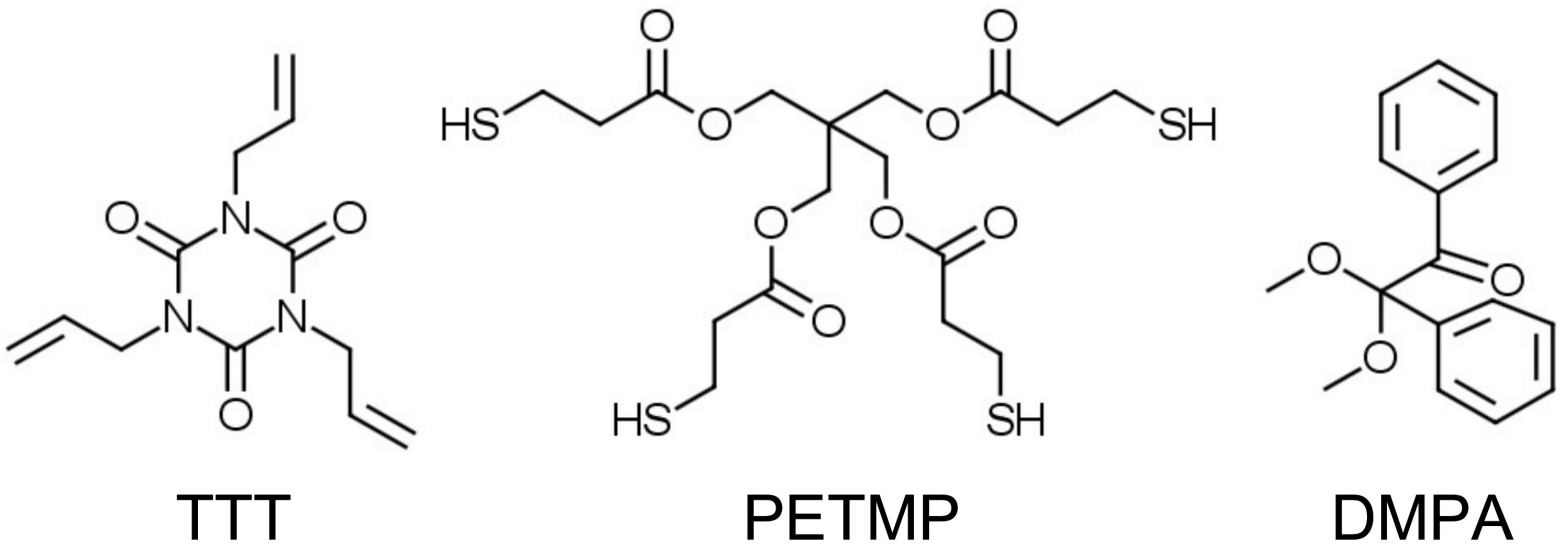
Chemical structures of the starting compounds. These structures can be also found via Sigma-Aldrich search engine. (TTT, trifunctional allyl component); (PETMP, tetrafunctional thiol component); and (DMPA, photoinitiator).

All reagents and solvents were obtained at the highest purity and used without further purification unless otherwise specified. For instance, 1,3,5-triallyl-1,3,5-triazine-2,4,6(1H,3H,5H)-trione (TTT, trifunctional allyl component), pentaerythritol tetrakis(3-mercaptopropionate) (PETMP, tetrafunctional thiol component), and 2,2-dimethoxy-2-phenylacetophenone (DMPA, photoinitiator) were obtained from Sigma-Aldrich. Chemical structures of the trifunctional allyl, tetrafunctional thiol components, and photoinitiator are shown in Scheme 3.

### 2.2. Preparation of the Thiol-Ene PETMP-TTT Networks

Photopolymerizable thiol-ene composition was prepared as a mixture of PETMP and TTT with 1:1 stoichiometric ratio of thiol to ene functional groups, containing 1 wt. % of DMPA. The reason we chose cleavage photoinitiator is that it gives higher quantum yield for the production of reactive radicals as compared to the hydrogen-transfer photoinitiators [20]. Photoinitiator was dissolved in a warm PETMP at 60 °C in an amber glass jar, then the calculated amount of TTT was added avoiding the direct day or artificial light; components were thoroughly mixed with a spatula. The clear colorless viscous mixtures were applied on flexible polyethylene terephthalate (PET) substrates (APLI paper S.A., product Ref. 10580) as a 100 µm thick layer via the Meyer rod coating method. The PET substrate (thickness 0.1 mm) side was suitable for inkjet printing and was used in the deposition process. The water contact angle (CA) for this substrate side was determined to be 30 ± 1°, which is significantly lower than for polydopamine-coated (CA = 49.8°) or carboxyl-group-modified (CA = 50.6°) PET [33], but close to the O_2_ plasma treated PET films (CA = 34 ± 1°) [34]. In another instance, deposition was performed on the polytetrafluoroethylene (PTFE) plate. Samples were cured simultaneously at the intensity of 1.64 mW/cm^2^ (wavelength: 254 nm) and 0.8 mW/cm^2^ (wavelength: 365 nm). After that, cured network was obtained, denoted as PETMP-TTT. Free-standing PETMP-TTT films were obtained by gently peeling the film from the PTFE plate.

### 2.3. Characterization

A Vertex 70 Fourier transform infrared (FTIR) spectrometer (Bruker Optics Inc., Ettlingen, Germany) equipped with a 30Spec (Pike Technologies) specular reflectance accessory having a fixed 30° angle of incidence (3/16″ sampling area mask), was used to record the spectra. The sample was laid face down across the top of the 30Spec accessory and the spectrum of the sample was recorded at a resolution of 4 cm^−1^. The software OPUS 6.0 (Bruker Optics Inc.) was used for data processing of the baseline correction of spectra. 

Differential scanning calorimetry (DSC) measurements were carried out by using a Q2000 thermosystem (TA Instruments). The samples were examined at a heating/cooling rate of 10 °C/min under nitrogen atmosphere. Thermogravimetric analysis (TGA) was performed on a Q50 analyzer (TA Instruments). The heating rate was 10 °C/min under nitrogen atmosphere.

The pencil hardness test was used to measure the hardness of the PETMP-TTT coating, according to the standard ASTMD 3363. A vertical load of 750 g was applied at an angle of 45° to the horizontal coating surface, as the pencil was moved over the sample. The grade of coating was judged by the worn surfaces immediately after the pencil hardness tests.

Optical properties of the PETMP-TTT coatings were evaluated by measuring ultraviolet–visible (UV–Vis) transmission. Measurements were conducted using a fiber optic UV/VIS/NIR Spectrometer AvaSpec-2048 (Avantes, Apeldoorn, the Netherlands) in the wavelength range from 300 to 800 nm, with a resolution of 1.4 nm.

Thermo-mechanical cyclic tensile testing was performed using machine H10KT (Tinius Olsen, Kongsberg, Norway) equipped with a temperature controllable chamber. The sample was first heated to 70 °C for 120 s and then strained to 1.0% at a speed of 20 mm/min. After that, the sample was cooled to room temperature while 1.0% of the strain was kept for 10 min to fix temporary elongation. Next, the lower clamp returned to the original position. Once the force on the sample was released, it was heated again to 70 °C in order to recover; that is when the second cycle started. This cycle was repeated three times and the stress-strain curves were recorded for analysis. The maximum strain in the cyclic tensile tests and the residual strain after recovering in the Nth cycle were determined from stress-strain curves and used to calculate the strain recovery ratio *R_r_*, as well as the total strain recovery ratio *R_r,tot_* after N passed cycles [35]. For the equations of *R_r_* and *R_r,tot_* (i.e. Equations (1) and (2)), please refer to [35]. 

Scratch testing of PETMP-TTT coatings was performed with a custom-made PC controlled scratch testing apparatus. During the scratch test the PETMP-TTT coatings were scratched (scratch length 10 mm and speed 0.2 mm/s) with a sphero-conical stylus (cone angle 90° and indenter radius 45 μm) applying the constant loading of 1.2 N, 1.5 N, and 2.7 N, respectively. The scratches were performed in air atmosphere (temperature 23 °C and humidity 40%). The B-600MET series upright metallurgical microscope (OPTIKA Srl, Ponteranica, Italy) with a c-mount 2560 × 1920 resolution (5.0 Mpixel) camera (Optikam Pro 5LT) was used for the inspection of a scratch track before and after thermal treatment (70 °C for 5 min) of the coating. Inspection was performed immediately after the scratch test. The optical images of the scratch tracks were converted to greyscale with brightness and contrast levels equalized for each image, respectively. Scratch track profiles were obtained using a precision surface roughness tester TR200 (SaluTron Messtechnik GmbH, Frechen, Germany). In another instance, sphero-conical stylus was replaced with 19 mm precision stainless steel miniature wire cup brush with 1/8 inch shank and the surface of the coating was brushed applying the constant loading of 1.2 N and speed of 0.2 mm/s for two cycles (i.e. forward and backwards = 1 cycle). Brushing track length was 10 mm. Afterwards, time-lapse optical microscopy inspection was performed in order to reveal the self-healing properties of PETMP-TTT coatings; no external stimulus for the coating was applied in this case.

Surface morphology of PETMP-TTT coatings was investigated using atomic force microscopy (AFM). AFM experiments were carried out at room temperature using a NanoWizardIII atomic force microscope (JPK Instruments, Bruker Nano GmbH, Berlin, Germany), while the data was analyzed using a SurfaceXplorer and JPKSPM Data Processing software (Version spm-4.3.13, JPK Instruments, Bruker Nano GmbH). The AFM images were collected using a V-shaped silicon cantilever (spring constant of 3 N/m, tip curvature radius of 10.0 nm and the cone angle of 20°) operating in AC mode.

## 3. Results and Discussion

**Figure 1:**
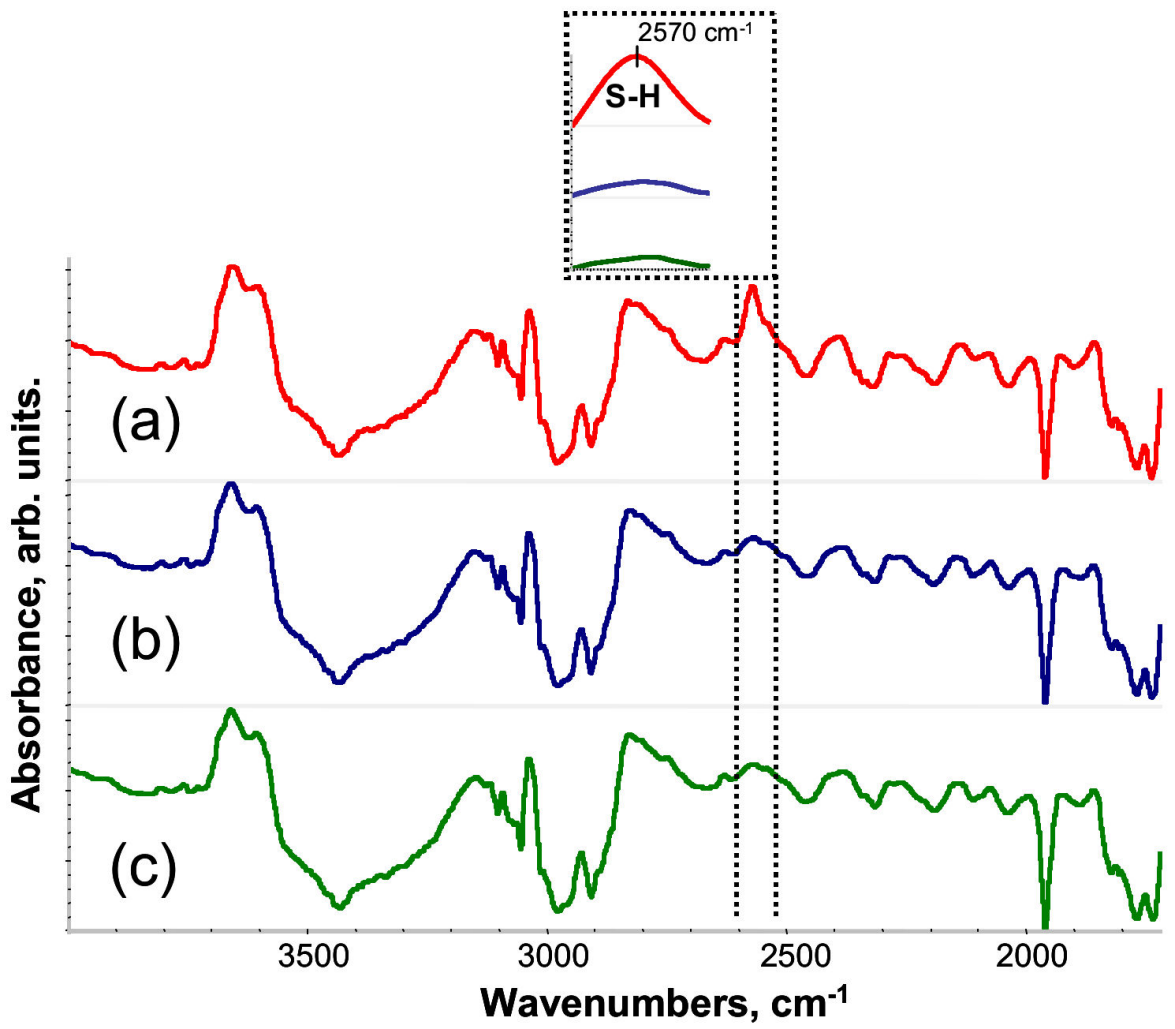
Fourier transform infrared (FTIR) spectra of PETMP-TTT reaction mixture for different UV curing time: (**a**) 0 s, (**b**) 30 s, and (**c**) 120 s. Inset: evolution of S–H band.

First, FTIR spectroscopy was used to determine optimal UV curing time for PETMP-TTT. Figure 1 shows the FTIR spectra of PETMP-TTT reaction mixture for the different UV curing time. The absorption peak of the S–H stretching band *v*_SH_ is located at 2570 cm^−1^ [36]. After 30 s, there was a significant decrease of *v*_SH_ (Figure 1b), indicating that thiol consumption proceeds very quickly. With the progress of the reaction, *v*_SH_ slightly decreases until UV-curing time is at 120 s. After that, no change of the *v*_SH_ intensity was observed, suggesting that the thiol conversion was complete. Accordingly, UV curing time of 120 s was chosen to form PETMP-TTT networks.

**Figure 2:**
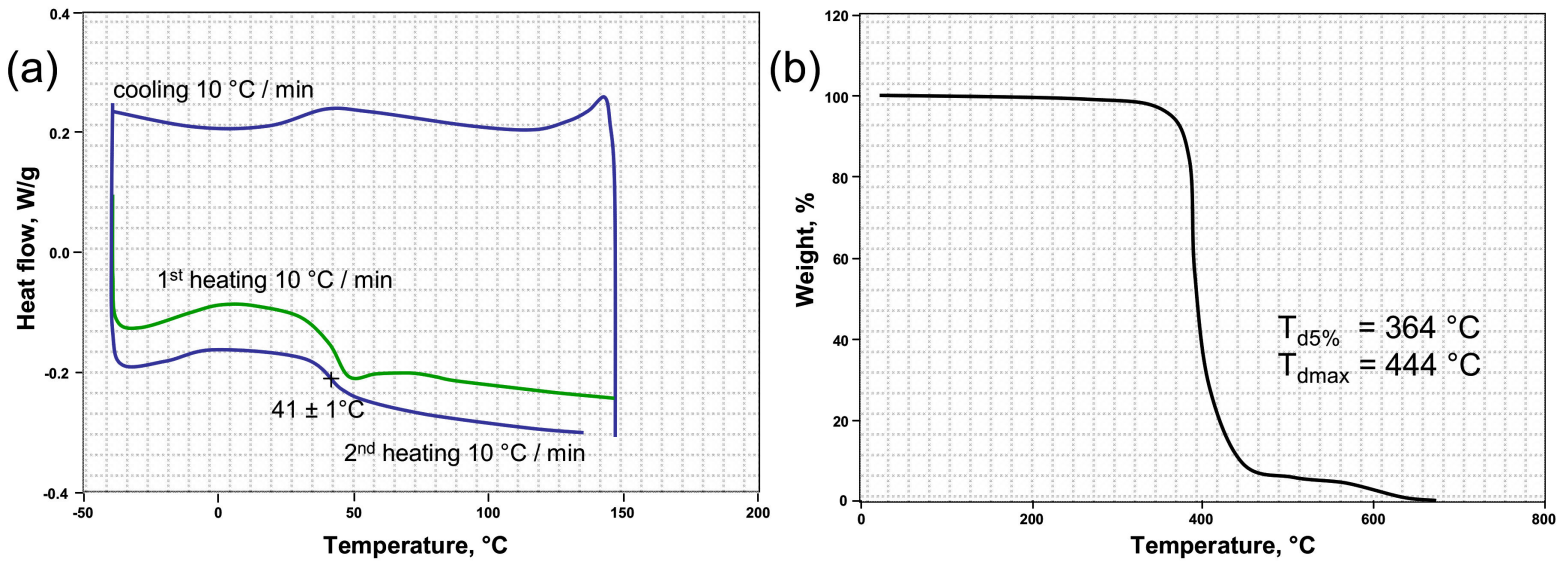
Differential scanning calorimetry (DSC) (**a**) and thermogravimetric analysis (TGA) (**b**) curves of the crosslinked PETMP-TTT polymer network.

Figure 2a shows the DSC curves of the crosslinked PETMP-TTT polymer network. The high thiol functionality and high conversion resulted in relatively high glass transition temperature (T_g_), with a value of 41 ± 1 °C and supported the FTIR results. This result is in good agreement with the T_g_ value previously reported in [37]. Thermal stability of the crosslinked PETMP-TTT polymer network was investigated by evaluating the weight loss behavior via TGA under nitrogen atmosphere. The quantitative properties such as the resultant weight loss temperature (T_d5%_) and the temperature corresponding to the maximum weight loss (T_dmax_) were determined from TGA curve and are shown in Figure 2b. It can be seen that PETMP-TTT polymer network exhibits high thermal stability with T_d5%_ = 364 °C, which can be attributed to high thiol functionality resulting in high crosslinking density in the polymer network, which is in agreement with the FTIR results. The high T_dmax_ (444 °C) shows that the crosslinked polymer network has a very good heat resistance. It may originate from the nitrogen heterocycle in PETMP-TTT system.

**Figure 3:**
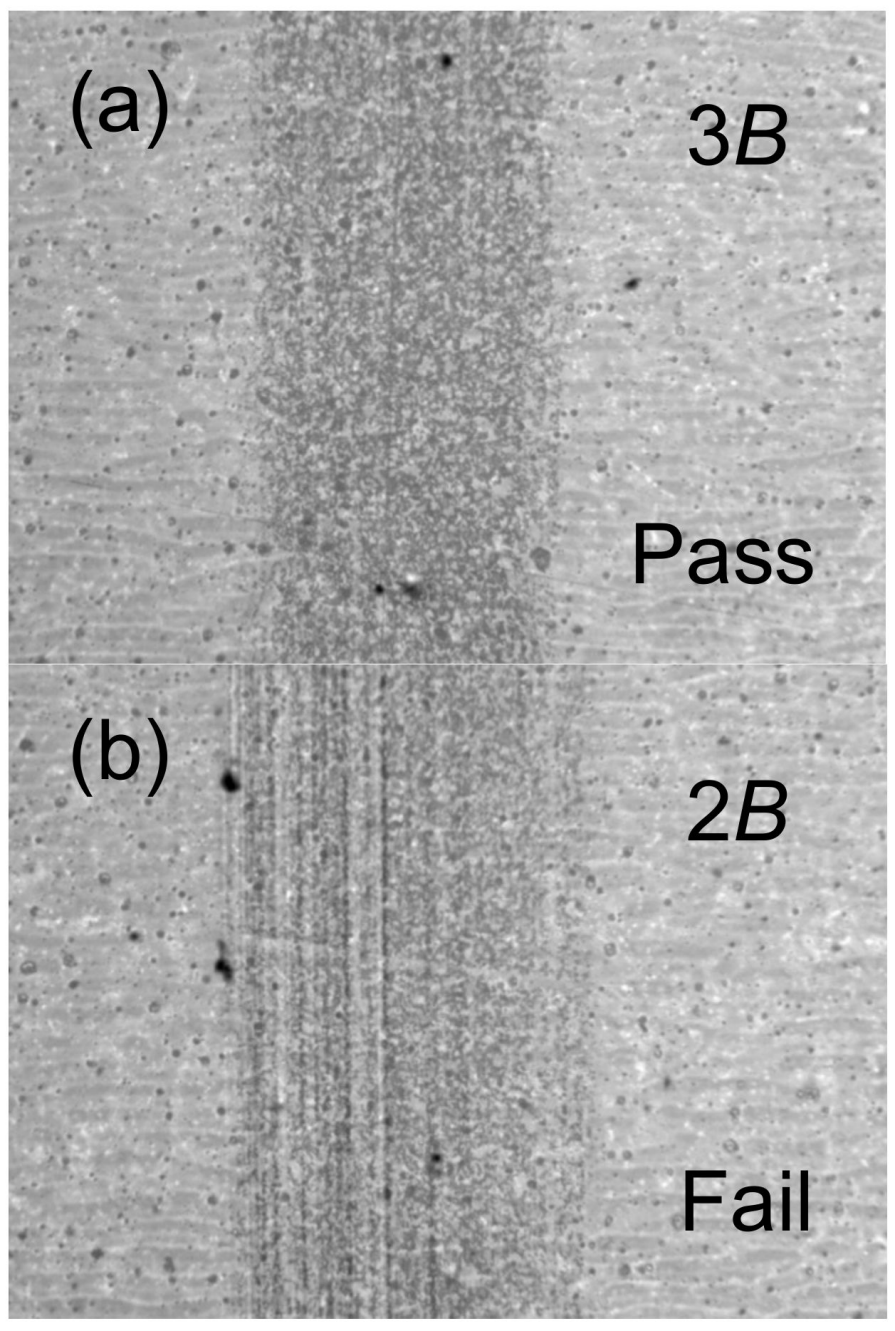
Characteristic optical microscope digital photographs (magnification 150×) of PETMP-TTT coatings after pencil hardness test: (**a**) 3B passed test and (**b**) 2B failed test.

Fabricated PETMP-TTT coatings could be characterized as glossy transparent films that ensure a smooth, comfortable touch feeling. The surface hardness of cured PETMP-TTT coatings was measured using pencil hardness test. Figure 3 shows the characteristic optical microscope digital photographs of PETMP-TTT coatings after the pencil hardness test. It was found that the pencil hardness value of 3B left no scratches on the coating surface—successfully passing the test—while the pencil hardness value of 2B failed. Shin, Junghwan, et al., who fabricated thiol-isocyanate-ene ternary networks by the sequential dual cure system, have also reported on similar pencil hardness value for polymer network with SH/C=C/NCO functional group molar ratio of 100:100:0 [38].

**Figure 4:**
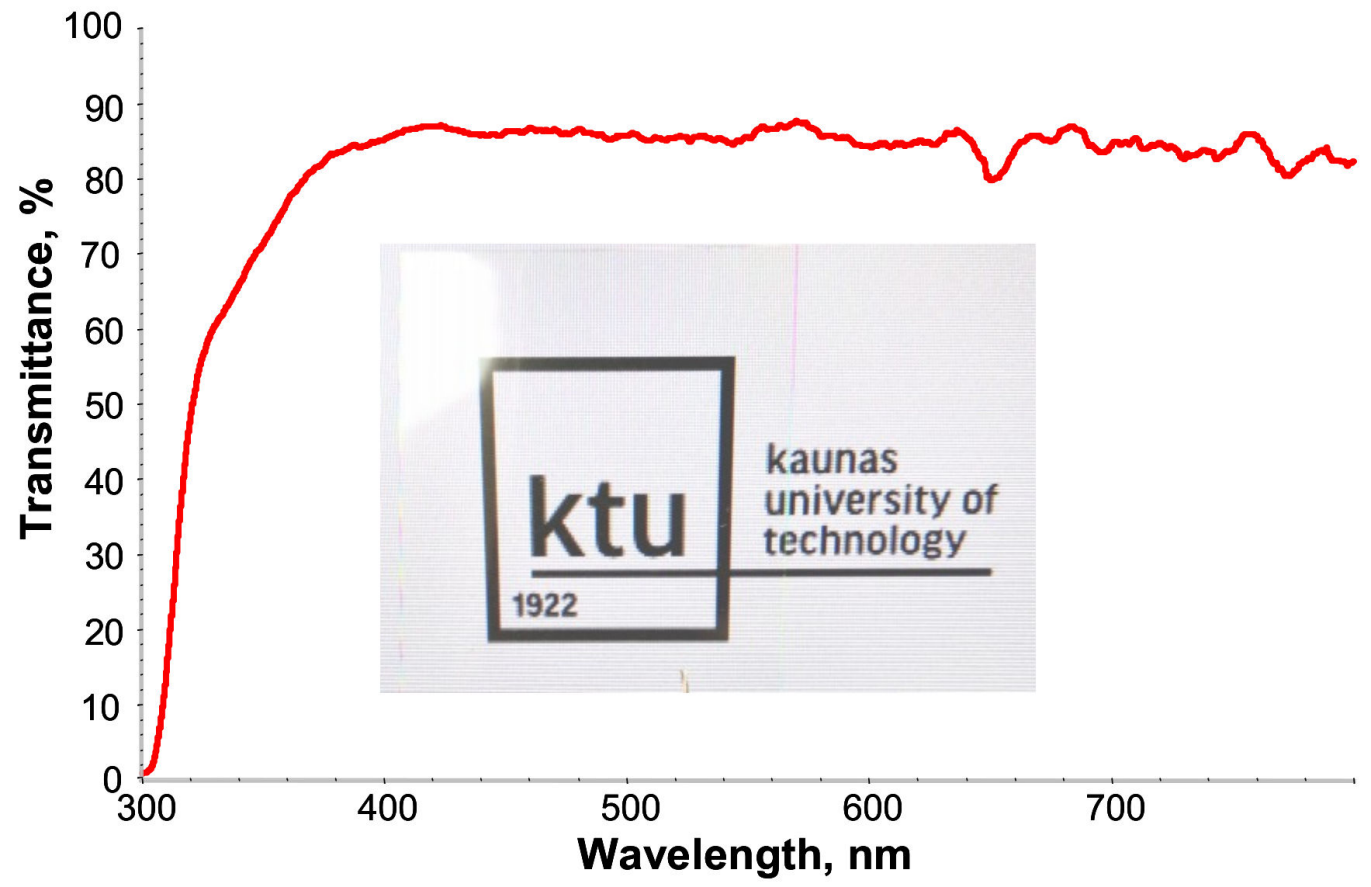
UV-vis spectrum and digital photo of PETMP-TTT coating on PET substrate over KTU logo, displayed through a computer monitor.

From the digital photo (Figure 4), it can be directly seen that the crosslinked PETMP-TTT polymer network has very good transparency. Specifically, PETMP-TTT coatings show high transmittance in the wavelength range of 400–800 nm. At 300 nm, the transmittance of PETMP-TTT polymer network on PET substrate is close to zero, indicating very good blocking effect on medium and short-wave UV light (UVB and UVC bands).

**Figure 5:**
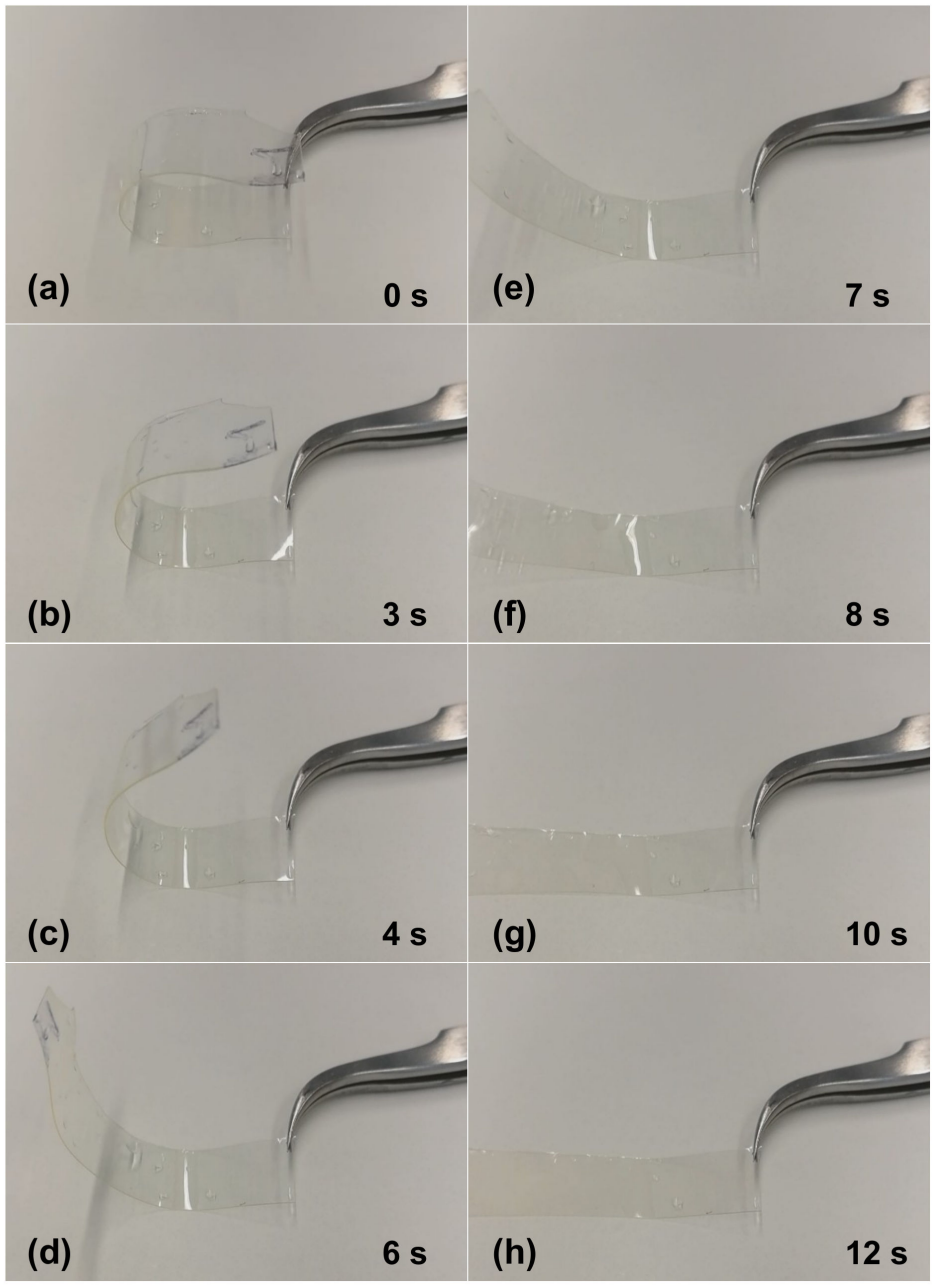
Shape-memory test of the crosslinked PETMP-TTT polymer free-standing film: (**a**) Digital photograph of free-standing PETMP-TTT film with fixed shape after cooling down below 0 °C and (**b**), (**c**), (**d**), (**e**), (**f**), (**g**), and (**h**) shape recovery process at different time instances when heated with hot air.

In order to verify shape-memory effect in the fabricated PETMP-TTT polymer film, a simple test was performed. Free-standing PETMP-TTT film was folded to a certain shape and while this shape was fixed, the PETMP-TTT polymer was cooled down below 0 °C and left to stand at room temperature for 5 min. Afterwards, a free-standing PETMP-TTT film was heated with hot air (50–60 °C) and the shape recovery process was recorded. Figure 5 shows the shape recovery process of the free-standing PETMP-TTT film at different time instances and when heated with a hot air. Before the heating started, the vitrified film “remembered” its temporary shape (Figure 5a) so that only minor instantaneous recovery could occur at an ambient temperature. Heating above *T_g_* triggered a fast return from temporary to the permanent shape through mobilization of the constituent polymer chains and release of latent strain energy. Full shape recovery of the tested film was achieved within 10 to 12 s. This result clearly indicated that crosslinked PETMP-TTT polymer network possesses shape-memory properties.

**Figure 6:**
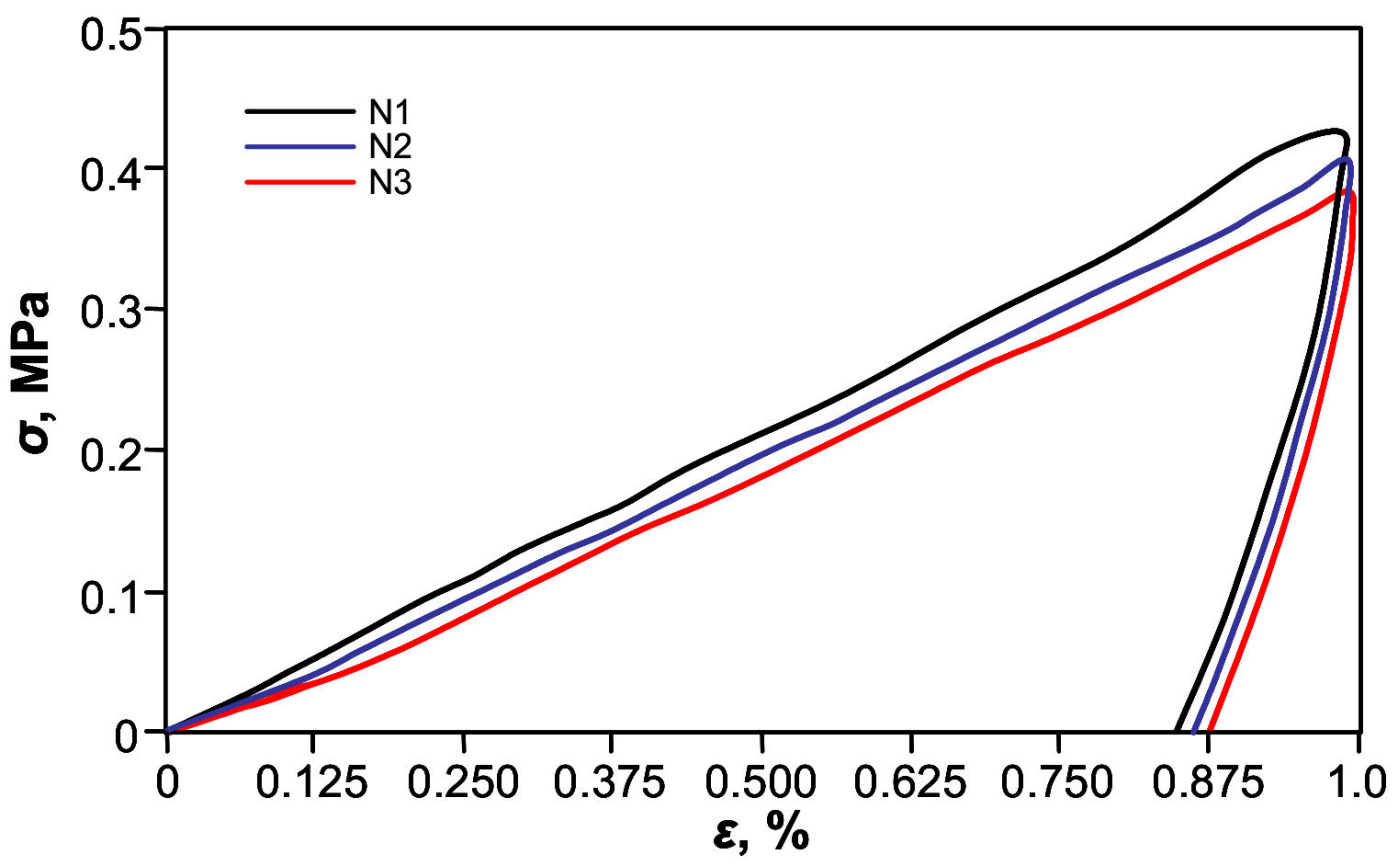
Cyclic tensile stress-strain curves of the crosslinked PETMP-TTT polymer network.

For further quantification of shape-memory’s effect on the thermo-mechanical cyclic, tensile testing was performed. Figure 6 shows shape-memory behavior of the free-standing PETMP-TTT film, with characteristic cyclic tensile stress-strain curves. The *R_r_* and *R_r,tot_* values for PETMP-TTT polymer were found to be better than 94 ± 1% and 97 ± 1%, respectively, which indicates very good shape-memory behavior comparable with recently published results for PEG blends [39]. A slight improvement of *R_r_* was observed with increase in the number of applied cycles: *R_r_* increased from 94 ± 1% (first cycle) to 96 ±1% after three cycles of the tensile test. It can be considered as a normal process for cyclic thermo-mechanical investigations, where the first few cycles often tend to differ from each other because of the history of the film and reorganization of the polymer chains [40].

**Figure 7:**
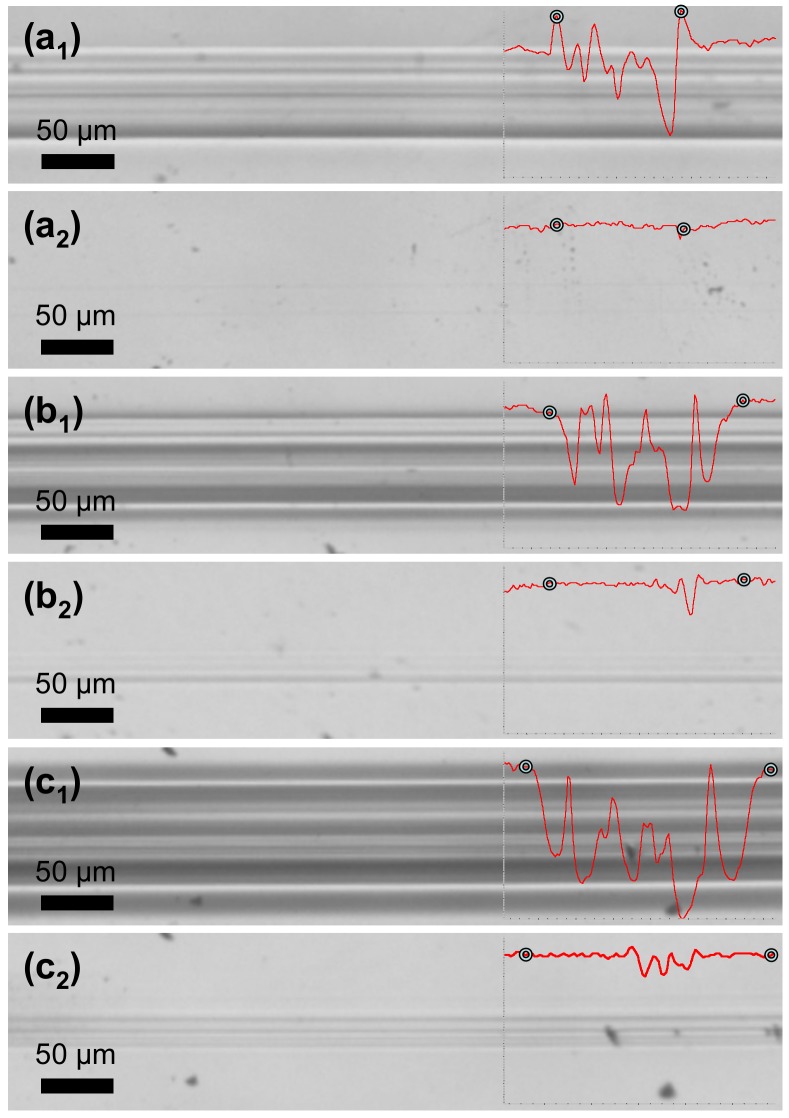
Scratch-healing of the crosslinked PETMP-TTT polymer coatings: (**a_1_**), (**b_1_**), and (**c_1_**) optical microscope digital photographs of characteristic scratch track sections before, and (**a_2_**), **(b_2_**), and (**c_2_**) after the healing process of the scratch with different constant loading of 1.2 N, 1.5 N, and 2.7 N, respectively. Insets show characteristic scratch track profiles with corresponding indicators for the beginning and the end of scratch.

Scratch testing was performed in order to investigate the scratch-healing properties of PETMP-TTT coatings after external stimulus was applied. Figure 7 shows optical microscope digital photographs of scratch tracks obtained on the coating with different constant loading as well as corresponding healing process of the scratch after 5 min at 70 °C. A scratch track profile area was used to calculate a scratch-healing ratio (*S_r_*) of PETMP-TTT coatings in Equation (1)

(1) $$S_{r}=\frac{A_{1}-A_{2}}{A_{1}}\times 100\%$$

where $A_{1}$ stands for the scratch track profile area after scratch, while $A_{2}$ stands for the scratch track profile area after healing. Indicators for the beginning and the end of scratch are added along the profiles. Scratch tracks obtained with the constant loading of 1.2 N and 1.5 N were almost completely healed after the thermal treatment of the PETMP-TTT coating with an *S_r_* of 96 and 94%. It is important to note that no decomposition of polymer was observed during the thermal treatment process. Furthermore, the crosslinked PETMP-TTT network also exhibited considerable scratch-healing (*S_r_* = 91%) properties of the scratches obtained with a higher constant loading of 2.7 N, as observed in Figure 7c_1_ and c_2_.

**Figure 8:**
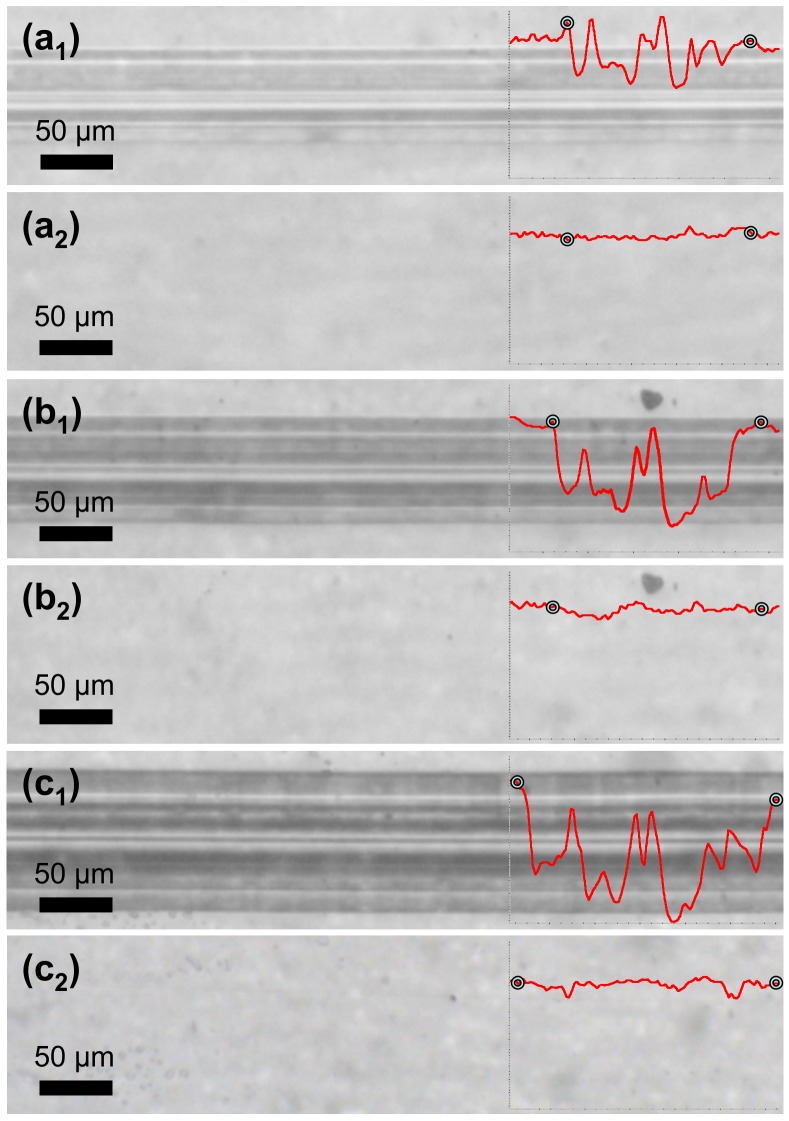
Shape-memory assisted scratch-healing of the quenched PETMP-TTT polymer coatings: (**a_1_**), (**b_1_**), and (**c_1_**) optical microscope digital photographs of characteristic scratch track sections before, and (**a_2_**), (**b_2_**), and (**c_2_**) after the healing process of the scratch with different constant loading of 1.2 N, 1.5 N, and 2.7 N, respectively. Insets show characteristic scratch track profiles with corresponding indicators for the beginning and the end of scratch.

In another instance, PETMP-TTT coatings were cooled down below 0 °C and left to stand at a room temperature for 5 min prior to the scratch testing. This procedure was performed in order to immobilize the polymer chains in covalently bonded 3D network and ensure improved form fixity. The testing procedure was analogous to the previously described scratch test. Figure 8 shows optical microscope digital photographs of scratch tracks obtained on the quenched coatings with different constant loading as well as corresponding healing process of the scratches after 5 min at 70 °C. It is evident that cooling down prior to the scratch testing resulted in a more efficient shape recovery of the scratched films. In all cases, the scratches were completely healed with a *S_r_* value of 99%. It is suggested that a quenching of the polymerized PETMP-TTT network serves as a freezing process for the internal strain energy, which is the primary driving force for the shape recovery of the scratched polymeric films.

**Figure 9:**
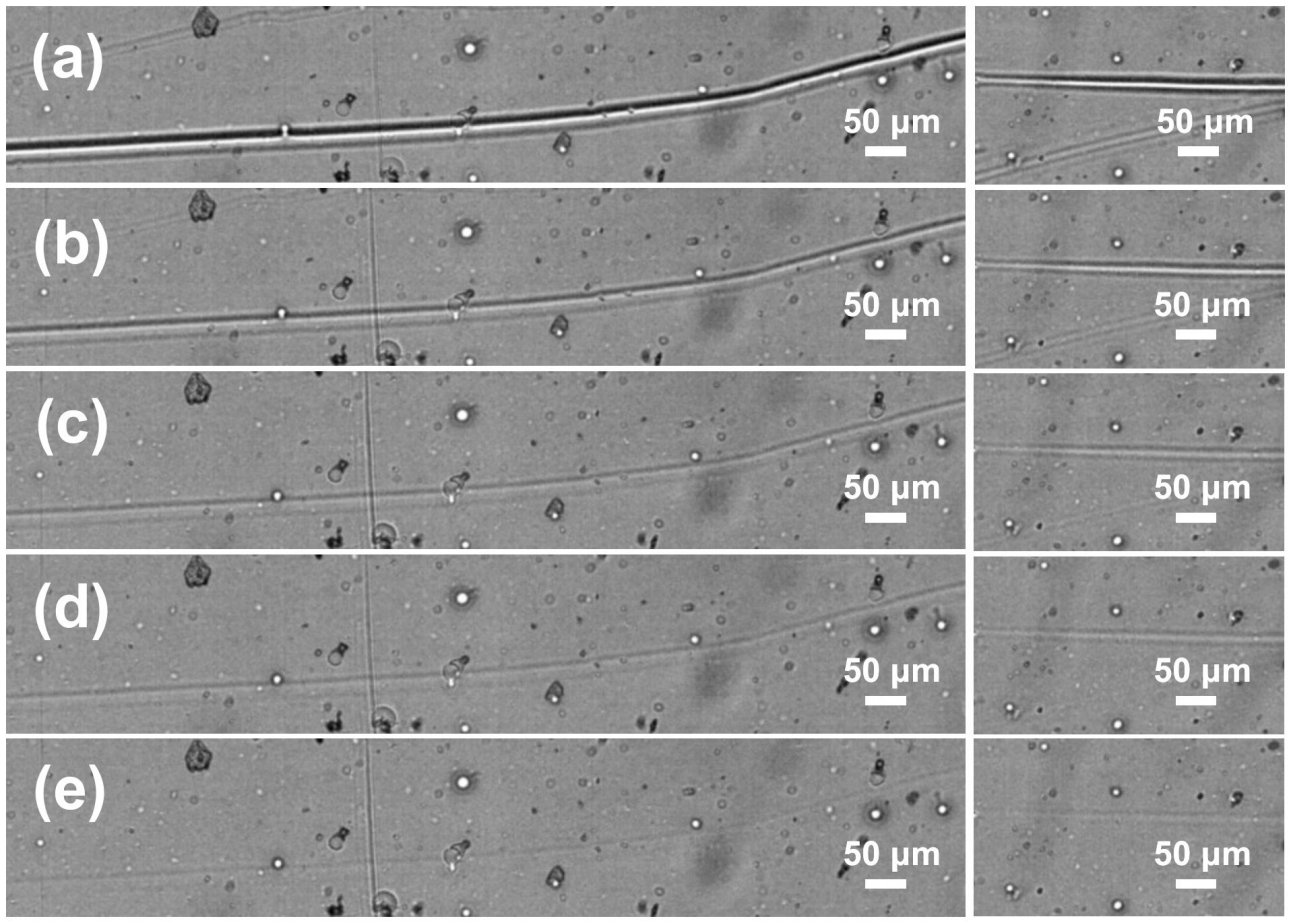
Scratch-healing of the crosslinked PETMP-TTT polymer network: (**a**) initial scratches obtained after brushing; (**b**), (**c**), (**d**), and (**e**) images of the scratches after 10 min, 30 min, 50 min, and 110 min at room temperature, respectively.

Further, the scratch self-repairing ability of PETMP-TTT coatings was investigated at room temperature and without external stimulus applied. In this case, the surface brushing tests with a stainless-steel miniature wire brush were performed. Figure 9 presents evolution of the initial film scratches with a time at ambient temperature. It can be seen that noticeable scratches were made by the brush wires having lateral dimensions of the order of tens of micrometers almost completely disappear within 110 min time span even at a room temperature. Although the PETMP-TTT coatings, that are heated above the T_g_, initiate the shape-memory actuation response more rapidly, the healing process for the brushed specimens is also quite effective. It can be associated with delayed elasticity of the distorted polymer network. When PETMP-TTT polymer is heated above the T_g_, the segmental mobility of the network is higher [41], resulting in a faster self-healing process.

**Figure 10:**
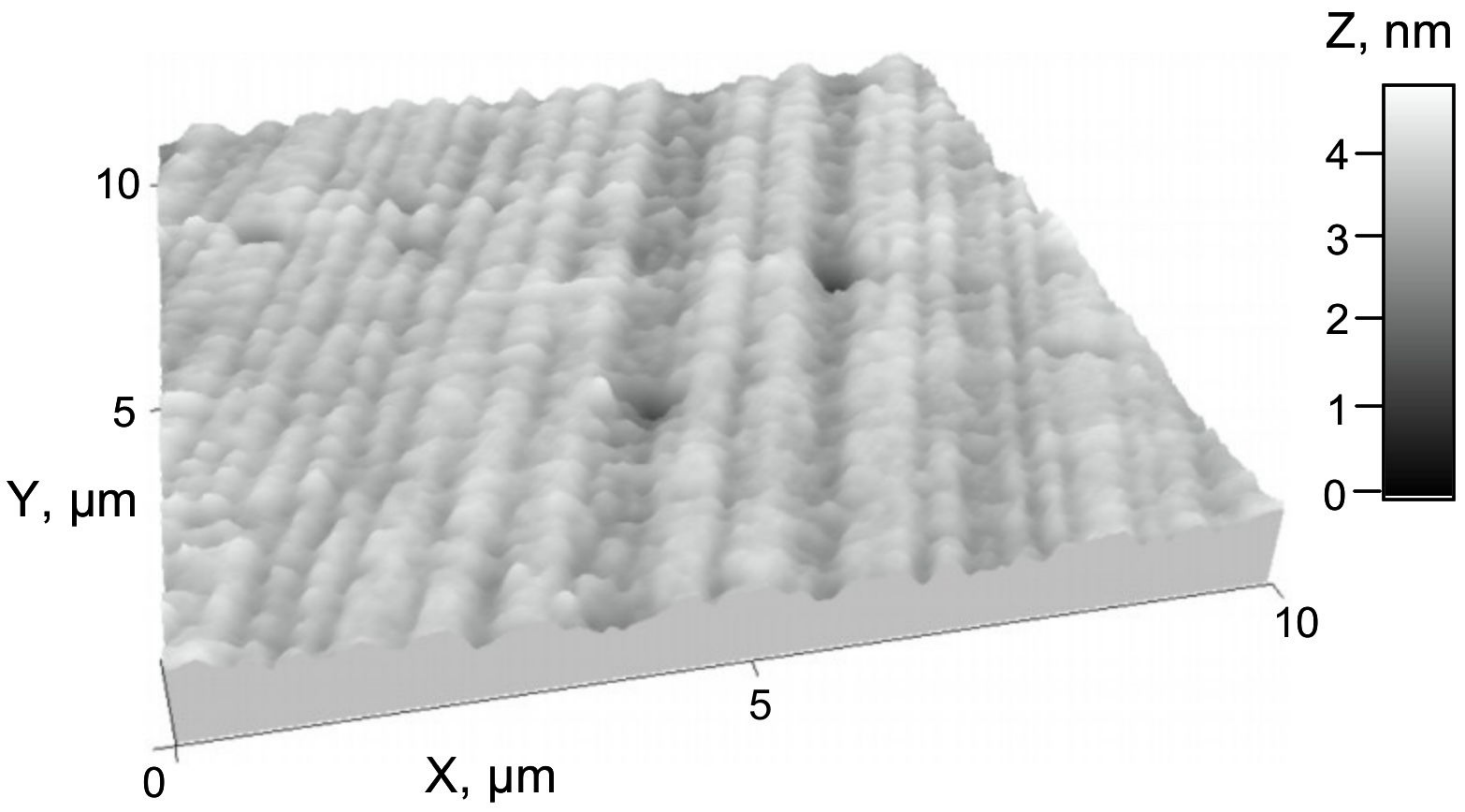
Characteristic atomic force microscopy (AFM) topographical image with normalized Z axis in nm of PETMP-TTT polymer coating.

AFM measurements were performed to investigate the surface morphology of PETMP-TTT polymer coating at the nanoscale (Figure 10). The topography of PETMP-TTT shows a homogeneous surface with 22.0 ± 2° (i.e., the most frequent orientation) oriented morphological features having a mean height of 3.64 nm and the root mean square roughness (*R_q_*) of 0.40 nm. The surface of PETMP-TTT is dominated by the valleys with skeweness (*R_sk_*) value of -0.93 and has a leptokurtoic distribution of the morphological features with kurtosis (*R_ku_*) value of 5.76, indicating many high peaks and low valleys.

**Figure 11:**
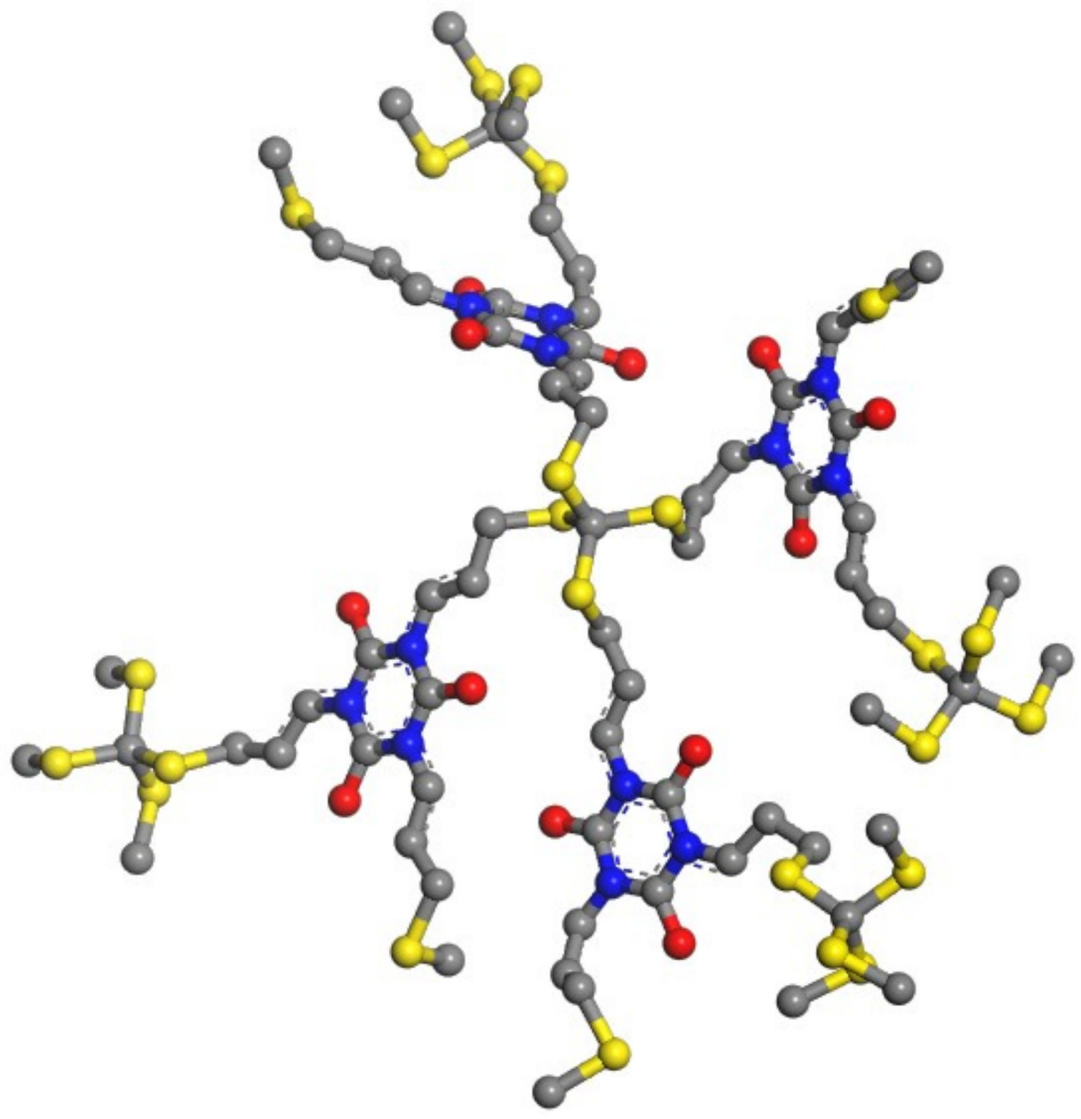
Representation of the crosslinked PETMP-TTT polymer network fragment.

With the emergence of new optoelectronic products like foldable smartphones and wearable electronic sensors with the flexible electronic components, scratch-healing properties of the materials acting as protective coatings will be more and more desirable. The particular crosslinked PETMP-TTT polymer network (Figure 11) that exhibits high optical transparency and efficient shape-memory assisted scratch-healing properties could be considered as a functional layer for the structured optoelectronic devices. Importantly, this is a significant finding, as the scratch-healing properties for PETMP-TTT polymer network were not explored previously. Synthesis, deposition, and curing technological procedures of PETMP-TTT coatings are not sophisticated, time efficient, and scalable, and thus could be easily integrated in the large area flexible electronic manufacturing processes.

## 4. Conclusions

The PETMP and TTT, acting as tetrafunctional thiol and trifunctional allyl components, respectively, were used in the preparation of photopolymerizable thiol-ene composition, with 1 wt. % of DMPA photoinitiator. An optimal UV-curing time of 120 s was determined using FTIR spectra analysis, and in particular, monitoring the intensity change of an S─H stretching band at 2570 cm^−1^. The crosslinked PETMP-TTT polymer network exhibited a relatively high glass transition temperature (T_g_) with a value of 41 ± 1 °C, indicating high thiol functionality and high conversion. TGA measurements showed that PETMP-TTT polymer network exhibits a high thermal stability, with T_d5%_ = 364 °C, which is a result of the high crosslinking density in the polymer network. The PETMP-TTT coatings passed the 3B pencil hardness test. The shape-memory test revealed that PETMP-TTT polymer network possesses shape-memory properties. Further quantification of the shape-memory behavior of the crosslinked PETMP-TTT polymer was obtained using cyclic tensile testing. The determined *R_r_* and *R_r,tot_* values were found to be better than 94 ± 1% and 97 ± 1%, respectively. It was found that scratches produced with a different constant loading of 1.2 N, 1.5 N, and 2.7 N on the PETMP-TTT coating heal more efficiently with a quenching procedure applied prior to the scratch testing. In this case, the scratches were completely healed with *S_r_* value of 99% for the all scratch testing instances. It was also found that the crosslinked PETMP-TTT polymer network was also capable to initiate a scratch recovery at ambient temperature conditions, presumably due to the delayed elasticity of the distorted polymer network. It was suggested that a crosslinked PETMP-TTT polymer network could be integrated as a scratch-healing and transparent coating in a new generation of the optoelectronic products.
